# Comparative Proteomic Analysis Reveals the Ascorbate Peroxidase-Mediated Plant Resistance to *Verticillium dahliae* in *Gossypium barbadense*

**DOI:** 10.3389/fpls.2022.877146

**Published:** 2022-05-19

**Authors:** Tianxin Lu, Liping Zhu, Yuxuan Liang, Fei Wang, Aiping Cao, Shuangquan Xie, Xifeng Chen, Haitao Shen, Beini Wang, Man Hu, Rong Li, Xiang Jin, Hongbin Li

**Affiliations:** ^1^Key Laboratory of Xinjiang Phytomedicine Resource and Utilization of Ministry of Education, Key Laboratory of Oasis Town and Mountain-Basin System Ecology of Xinjiang Production and Construction Corps, College of Life Sciences, Shihezi University, Shihezi, China; ^2^Research Center for Wild Animal and Plant Resource Protection and Utilization, Qiongtai Normal University, Haikou, China; ^3^Ministry of Education Key Laboratory for Ecology of Tropical Islands, Key Laboratory of Tropical Animal and Plant Ecology of Hainan Province, College of Life Sciences, Hainan Normal University, Haikou, China

**Keywords:** *Gossypium barbadense*, *Verticillium dahliae*, comparative proteomics, reactive oxygen species, ascorbate peroxidase

## Abstract

In previous research on the resistance of cotton to Verticillium wilt (VW), *Gossypium hirsutum* and *G. barbadense* were usually used as the susceptible and resistant cotton species, despite their different genetic backgrounds. Herein, we present data independent acquisition (DIA)-based comparative proteomic analysis of two *G. barbadense* cultivars differing in VW tolerance, susceptible XH7 and resistant XH21. A total of 4,118 proteins were identified, and 885 of them were differentially abundant proteins (DAPs). Eight co-expressed modules were identified through weighted gene co-expression network analysis. GO enrichment analysis of the module that significantly correlated with *V. dahliae* infection time revealed that oxidoreductase and peroxidase were the most significantly enriched GO terms. The last-step rate-limiting enzyme for ascorbate acid (AsA) biosynthesis was further uncovered in the significantly enriched GO terms of the 184 XH21-specific DAPs. Additionally, the expression of ascorbate peroxidase (*APX*) members showed quick accumulation after inoculation. Compared to XH7, XH21 contained consistently higher AsA contents and rapidly increased levels of *APX* expression, suggesting their potential importance for the resistance to *V. dahliae*. Silencing *GbAPX1*/*12* in both XH7 and XH 21 resulted in a dramatic reduction in VW resistance. Our data indicate that APX-mediated oxidoreductive metabolism is important for VW resistance in cotton.

## Introduction

*Verticillium dahliae* Kleb is the fungal pathogen of Verticillium wilt (VW) that commonly causes dramatic reductions in the production of crops such as cotton, tomato, and tobacco (Song et al., 2020). *V. dahliae* was first reported in Virginia, United States, in 1914 and spread to many cotton-producing regions in China during the 1930s (Shaban et al., 2018). To date, more than half of the cotton fields in China contain *V. dahliae* pathogen and VW can lead to 30–50% yield reduction, sometimes even causes total yield loss (Zhang et al., 2020). VW usually causes more severe damage in *G. hirsutum* than in *G. barbadense* (Ma et al., 1999). Little progress has been made in cotton breeding for VW resistance, either in *G. hirsutum* or in *G. barbadense* (Liu et al., 2018b).

The virulence mechanism exhibited by *V. dahliae* is predominantly induced through propagation in the vascular system, and finally leads to xylem vessel blockage, resulting in severe leaf chlorosis and wilting, leaf and boll abscission, and even plant death (Klosterman et al., 2009). For decades, efforts have been made by researchers to investigate the molecular mechanisms of VW-defense in cotton. It has been demonstrated that the resistance of cotton to VW primarily depends on preformed defense structures, such as thick cuticles, accumulation of phenolic compounds and structures delaying or hindering the expansion of the invader (Shaban et al., 2018). The proteins that are responsible for the resistance of cotton to *V. dahlia* have been identified, and these proteins include immune-related proteins, receptor-like kinases, and transcription factors, such as apoplastic thioredoxin protein (GbNRX1), the receptor-like kinase suppressor of BIR1-1 (GbSOBIR1) and MYB transcription factors (GhMYB108) (Cheng et al., 2016; Li et al., 2016; Zhou et al., 2019). Proteins that play various roles in cell wall modification and/or development, such as proline-rich protein GbHyPRP1 (which can thicken cell walls), are also involve in VW resistance (Yang et al., 2018). When the lignification of cell walls is increased and pectin methylesterase is inhibited, the resistance to VW is enhanced (Liu et al., 2018a). Furthermore, researchers have even identified cotton proteins that can directly degrade chitin in fungal cell walls to facilitate immune recognition (Han et al., 2019).

Reactive oxygen species (ROS) are important signaling molecules that have significant roles in plant development, signal transduction and environmental stress responses (Mittler et al., 2004; Li et al., 2007). Hydrogen peroxide (H_2_O_2_) is the major form of ROS in plants and is mainly produced in peroxisomes, chloroplasts and mitochondria; in addition, a high content of H_2_O_2_ in apoplast, which is the extracellular space between the plasma membrane and cell wall, is toxic to plant cells (Smirnoff and Arnaud, 2019). Higher plants have at least four types of peroxidases, glutathione peroxidases (GPX), catalase (CAT), ascorbate peroxidase (APX, class I peroxidase, intracellular) and plant-specific class III peroxidase (Prx, secreted) (Hiraga et al., 2001). Numerous studies have shown that Prxs are involved in plant defense, mainly through the reinforcement of cell walls, ROS metabolism, and the production of anti-microbial metabolites (Passardi et al., 2004; Okazaki et al., 2007). It has been reported that redox homeostasis is important for the elongation of fiber in cotton (Guo et al., 2016; Tao et al., 2018). Moreover, ROS scavenging is also considered important for VW resistance in cotton; for instance, a novel cluster of glutathione S-transferase genes was reported to provide VW resistance in cotton (Li et al., 2019). An NBS-LRR protein from *G. barbadense* was also identified to enhance VW resistance in *Arabidopsis* through the activation of ROS production and the ethylene signaling pathway (Li et al., 2018). Thus, investigating the potential roles of APX (class I) and Prx (class III) peroxidases in cotton resistance to VW will improve our understanding of redox homeostasis in the plant pathogen response.

Proteomics is frequently used for investigations on VW resistance in various plants and provides useful information for understanding the molecular mechanisms of disease resistance (Wang et al., 2018; Hu et al., 2019; Wu et al., 2019). In *V. dahliae*-inoculated *G. thurberi*, 6,533 proteins were identified in the roots, and salicylic acid was found to be significantly accumulated (Fang et al., 2015). Proteomics analysis of xylem sap in cotton showed that most of the over-accumulated proteins belonged to pathogenesis-related and cell wall proteins, while the under-accumulated and absent proteins were principally related to plant growth and development (Yang et al., 2020). Two-dimensional gel electrophoresis (2-DE)-based proteomic techniques have been applied for almost four decades since the 1980s, while liquid chromatography coupled to tandem mass spectrometry (LC–MS/MS) gel-free proteomic approaches have been predominant in recent years due to their high sensitivity and throughput (Roe and Griffin, 2006). Data-independent acquisition (DIA), an attractive MS analysis method, has recently emerged as a powerful approach for label-free relative protein quantification at the whole proteome level. With the DIA approach, thousands of proteins could be identified and quantified without performing fractionation, and only a few micrograms of the protein sample was needed (Pino et al., 2020).

Previous research on cotton VW resistance usually used *G. hirsutum* as a susceptible cotton species and *G. barbadense* as a resistant one, despite their different genetic backgrounds. To eliminate genetic background variation, we performed a DIA proteomics analysis of two *G. barbadense* varieties, susceptible XH7 and resistant XH21. A total of 4,118 proteins were identified, of which 885 proteins were differentially abundant proteins (DAPs) under the threshold of 1.5-fold change and *p* < 0.05. Weighted gene co-expression network analysis (WGCNA) showed that peroxidase activity was the most significantly enriched gene ontology term from the module that showed the most significant correlation with the time of fungal infection. In addition, one enzyme that is crucial for the biosynthesis of ascorbate acid (AsA) was observed in the most significantly enriched GO terms of XH21-specific DAPs. The expression levels of ascorbate peroxidase (*APX*) members were induced when the content of H_2_O_2_ increased during *V. dahliae* infection. Silencing *GbAPX1* and *GbAPX12* using virus-induced gene silencing (VIGS) in both XH7 and XH21 resulted in a dramatic reduction in VW tolerance. Our data provide the proteome profiles of *G. barbadense* varieties with different resistances to *V. dahliae* and reveal that the key members of the *APX* family are important for *V. dahliae* resistance in Pima cotton.

## Materials and Methods

### Cotton Material and Fungal Treatment

XH7 and XH21 cotton plants were cultured in sterilized soil in an artificial climate room under 70% humidity, 30°C and a 16/8 h light/dark cycle. Four-week-old seedlings were used for inoculation with *V. dahliae*. The *V. dahliae* strain V592 was activated using potato-agar medium and then grown on Czapek’s medium (30 g/L sucrose, 3 g/L NaNO_3_, 0.5 g/L MgSO_4_-7H_2_O, 0.5 g/L KCl, 100 mg/L FeSO_4_-7H_2_O, 1 g/L K_2_HPO_4_, pH 7.2) under 25°C for 5 days. Fungus spores were filtered using four-layer gauze to remove mycelium, and then the spore concentration was adjusted to 10^7^ per milliliter in liquid medium. The cotton seedlings were incubated with fungi at 25°C and shaken at 200 rpm for 50 min. The cotton seedlings were then transferred into Hoagland’s nutrient solution (Hoagland, 1920) for 3 weeks before phenotype identification. For high-throughput proteomic analysis, cotton roots from XH7 and XH21 were collected at 0, 6, and 24 h after incubation with *V. dahliae* and immediately frozen in liquid nitrogen before storage at –80°C. Three independent treatment replicates were performed for each time point.

### Protein Extraction and Liquid Chromatography Coupled to Tandem Mass Spectrometry

The roots from ten cotton plants were used for protein extraction using an improved protein extraction method as previously reported (Jin et al., 2019). Protein quantification was performed following the Bradford method (Bradford, 1976) using a UV-160 spectrophotometer (Shimadzu, Kyoto, Japan). After concentration determination, 100 μg of total protein from each sample was used for trypsin digestion as previously described (Jin et al., 2019). After digestion, iRT (Escher et al., 2012) and digested peptides were mixed in a 1:10 volume ratio. Then, samples were recovered in phase A [2% acetonitrile (ACN), pH 10] and injected into an Agilent 1100 HPLC system (Agilent Technologies, Santa Clara, CA, United States). The samples were then fractionated into 10 fractions using an Agilent Zorbax Extend-C18 column under a 50 min gradient of phase B (90% ACN, pH 10) with a 300 μL/min flow rate. The fractions were then vacuum freeze dried and subjected to the subsequent nanoLC–MS/MS experiment, which was carried out using a DIA method (Bruderer et al., 2017) on the orbitrap Fusion Lumos platform (Thermo Fisher Scientific, Rockford, IL, United States). Positive ion and high-resolution (120,000 resolution at m/z 200 with automatic gain control target of 3e^6^) modes were used for MS/MS data collection. The mass spectra scan range was set to 350–1,650 m/z. The isolation window for MS2 was set to 26 m/z, and the normalized collision energy was 28%.

DIA spectra were analyzed using Spectronaut pulsar 13.7.190916 (Bernhardt et al., 2012) against the protein database derived from the genome sequence of *G. barbadense* (Wang et al., 2019) with the following settings: missed cleavage, 2; fixed modification, carbamidomethyl; variable modification, oxidation; and protein FDR cut-off, 0.05. The DIA configuration was as follows: precursor *q*-value cut-off: 0.01; protein *q*-value cut-off: 0.01; normalization strategy: local normalization; and quantity MS-Level: MS2. Proteins that were observed in at least two out of three replicates were considered high-quality identified proteins. Proteins specifically found in only one cotton variety were defined as variety-specific proteins. For common proteins that could be observed in all samples, fold change ratios of over 1.5 with a *p*-value < 0.05 were considered DAPs.

### Bioinformatics Analyses

For further bioinformatic analyses, a heatmap was constructed using Heatmapper^1^ (Babicki et al., 2016). Protein co-expression network analysis was performed with the R package WGCNA as previously described (Langfelder and Horvath, 2008). The GO analysis of DAPs was performed using the Cytoscape plug-in ClueGO (Gabriela et al., 2009), while GO analysis for cotton variety-specific DAPs was carried out using agriGO 2.0 (Tian et al., 2017).

### RNA Extraction and Polymerase Chain Reaction

Total RNA was extracted from XH7 and XH21 cotton roots at 0, 6, and 24 h after a treatment with *V. dahliae* using an RNA extraction Kit (DP441, Tiangen, Beijing, China). cDNA was synthesized using a Takara reverse transcription Kit (K1622, Takara, Kusatsu, Japan). Semiquantitative polymerase chain reaction (PCR) was carried out using agarose gel electrophoresis by normalizing the housekeeping gene G*bUBQ*. Real-time quantitative PCR (qRT-PCR) was performed using SYBR green real-time PCR master premix (Applied Biosystems, Foster, CA, United States). The relative expression level of each tested gene was calculated using the 2^–△*Ct*^ method with *GbUBQ* set to 1 unless otherwise stated. All qRT–PCR results are shown as the mean ± SD from three independent biological replicates. The primers used in this work are provided in Supplementary Table 1.

### H_2_O_2_ and Ascorbate Acid Measurement

The content of H_2_O_2_ of cotton root was determined using a Micro Hydrogen Peroxide Assay Kit (BC3590, Solarbio, Beijing, China) and AsA was measured using an Ascorbic Acid Assay Kit (BC1230, Solarbio, Beijing, China) based on the methods in Wu et al. (2017).

### 3,3′-Diaminobenzidine Staining

DAB (3,3′-diaminobenzidine) staining of cotton leaves was performed according to Zheng et al. (2021). Briefly, cotton leaves were incubated in 1 mg/ml DAB-HCl, pH 3.8, in the dark for 8 h. The leaves were then cleared of pigment by boiling in an ethanol/acetic acid/glycerin mixture (3:1:1 v/v/v) for 20 min before imaging.

### Virus Induced Gene Silencing

A VIGS system (Burch-Smith et al., 2004) was used to validate the functions of *GbAPX1/12* in cotton *V. dahliae* tolerance. The conserved fragments of target genes were cloned into the pTRV2 vector (TRV:*GbAPX1/12*) using the *Asc*I and *Spe*I restriction sites. TRV:*GbCLA* was also constructed as a positive marker, in which white leaves are observed in gene silencing transformants. Empty vector TRV:00 was used as a negative control. All vectors were introduced into the Agrobacterium GV3101. After injection into cotton cotyledons, the plants were placed in the dark for 24 h before being exposed to normal growth conditions. After 2 weeks, the successful silencing of target genes was verified by qRT–PCR, and positive plants were selected for *V. dahliae* tolerance analyses.

### Statistical Analysis

All statistical analyses in this work were performed using SPSS 20.0 with one-way ANOVA and least significant difference methods. The asterisks represent statistical significance: *p < 0.05; ^**^p < 0.01.

## Results

### Phenotypes of XH7 and XH21 After Infection and Weighted Gene Co-expression Network Analysis of Differentially Abundant Proteins

**FIGURE 1:**
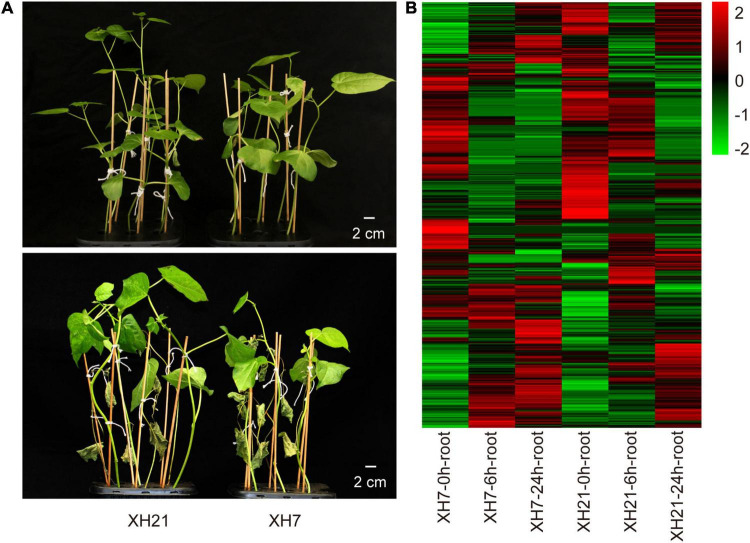
Symptoms of Verticillium wilt disease and heatmap of differentially abundant proteins (DAPs). **(A)** Representative seedlings of XH21 and XH7 before (upper panel) and 21 days after (lower panel) incubation with *Verticillium dahliae*. Four-week-old seedlings were infected with *V. dahliae* and were photographed after 3 weeks. *N* = 18 for each group. **(B)** Heatmap of 885 DAPs. The Log_2_ value of the expression levels of DAPs was used to produce the heatmap.

Compared to XH21, XH7 exhibited more severe disease symptoms with more wilting leaves and smaller plants, as well as higher disease indexes at 3 weeks after infection by *V. dahliae* (Figure 1A and Supplementary Figure 1). Total proteins of the XH7 and XH21 roots at 0, 6, and 24 h after fungal treatment were extracted, and DIA proteomics analysis was performed with three biological replicates each. The Venn diagrams of all replicates showed a high consistency among the three replicates (Supplementary Figure 2). A total of 4,118 proteins were identified with high confidence. Furthermore, the proteins with a signal intensity fold change of over 1.5 compared to that at 0 h separately in XH7 or XH21 were considered DAPs that responded to *V. dahliae* infection (Supplementary Figure 3).

A total of 885 DAPs were determined with the threshold of fold change over 1.5 and *p* < 0.05, which were then used for subsequent bioinformatic analyses (Figure 1B and Supplementary Table 2). Eight co-expression modules were observed for the WGCNA of all the DAPs. The turquoise (0.88) and blue (–0.8) modules showed the most positive and negative relationships with the time point, while the black (0.76) and red (–0.71) modules showed the most significant relationships with varieties (Supplementary Figure 4A). The interactions among these modules are shown in Topological Overlap Matrix (Supplementary Figure 4B), suggesting that the modules were relatively independent.

### Pathway Enrichment Analyses and Polymerase Chain Reaction Validation of the Module With the Highest Time Correlation and XH21-Specific Differentially Abundant Proteins

**FIGURE 2:**
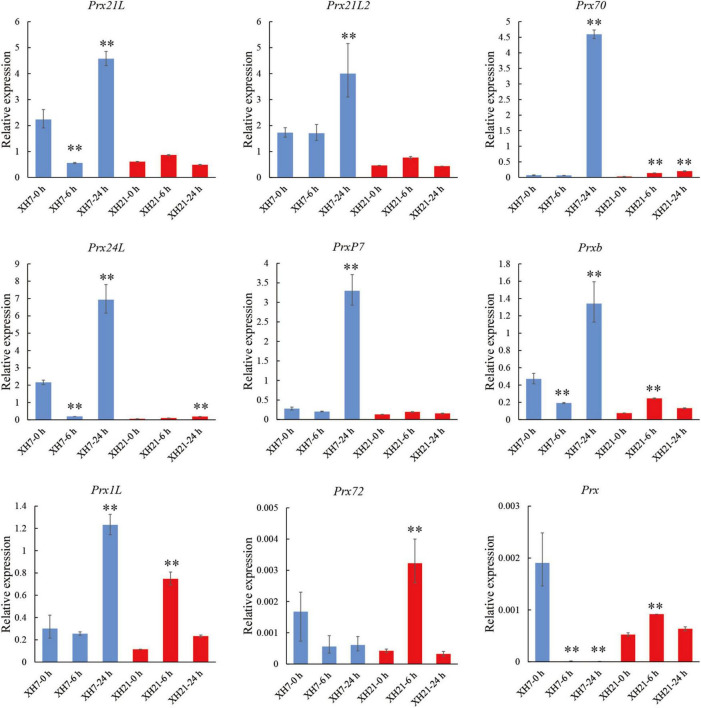
Transcriptional expression analysis of nine peroxidases. Relative expression levels of nine peroxidases identified in GO enrichment analysis. *GbUBQ* was used as an internal control and set to 1. Three independent replicates were performed for each PCR assay. ^**^p < 0.01.

To investigate the enriched pathways, DAPs from the turquoise module were then subjected to a Cytoscape plug-in ClueGo (Gabriela et al., 2009). For the biological process category, 255 DAPs of the turquoise module were enriched in three clusters, in which the GO terms of organonitrogen compound biosynthetic process, oxidoreductase activity and peroxidase activity were the most enriched GO terms (Supplementary Figure 5A). Moreover, intracellular non-membrane-bounded organelle, cytosol and chloroplast stroma were the most enriched GO terms for the cellular component category, while carbon-oxygen lyase activity, coenzyme binding and RNA binding were the most enriched for the molecular function category (Supplementary Figures 5B,C). We noticed that nine peroxidases were significantly enriched in the GO term peroxidase activity for both biological process and molecular function categories. Thus, qPCR assays were performed to validate whether the mRNA levels of these peroxidases changed. Of the nine peroxidases-coding genes, the expressions of *GbPrx72* and *GbPrx* were extremely low. Seven detectable genes showed significantly up-regulated mRNA levels after *V. dahliae* infection in both XH7 and XH21 (Figure 2). Together, the proteomics and qPCR data showed that the majority of class III peroxidases were significantly up-regulated at both the mRNA and protein levels after *V. dahliae* infection in both high- and low-susceptibility *G. barbadense* cultivars, indicating that extracellular redox homeostasis might be important for cotton *V. dahliae* resistance.

In addition to the common DAPs, this study also provided insights into the XH21-specific DAPs, which were probably responsible for the high tolerance to *V. dahliae* in XH21. A total of 184 XH21-specific DAPs were identified, and the detailed information is provided in Supplementary Table 3. Furthermore, GO enrichment analysis of the 184 XH21-specific DAPs was performed using software AgriGO. For the molecular function category, the significantly enriched end-terms of the tree-view were structural constituent of ribosome (GO:0003735), lyase activity (GO:0016829), protein heterodimerization activity (GO:0046982), RNA binding (GO:0003723) and coenzyme binding (GO:0050662) (Supplementary Figure 6). The end-terms for the biological process category were translation (GO:0006412) and glycolytic process (GO:0006069) (Supplementary Figure 7), while the end-terms for the cellular component category were ribosome subunit (GO:0044391) and nucleosome (GO:0000786) (Supplementary Figure 8). Through qPCR, we further examined the expression levels of nine genes that were enriched in the GO term coenzyme binding (GO:0050662). Seven genes exhibited mRNA expression levels that were consistent with the protein accumulation patterns between XH7 and XH21 (*GbYUCCA10*, *GbAcox1*, *GbDFR*, *GbPDC*, *GbAKHSD*, *GbPDC2*, and *GbGLDH*) (Supplementary Figure 9, gene names and primers are provided in Supplementary Table 1). Notably, one of the validated genes, *GbGLDH*, was considered the rate-limiting enzyme for the biosynthesis of AsA (Mellidou and Kanellis, 2017), which is one of the key metabolites that reduces H_2_O_2_ by the catalysis of ascorbate peroxidases (APXs). Collectively, we found that redox homeostasis-related proteins were significantly enriched in DAPs that were common to both XH7 and XH21, and XH21-specific accumulated proteins.

### Ascorbate Acid and H_2_O_2_ Contents and the Expression of Ascorbate Peroxidases Are Important for *Verticillium dahliae* Resistance in Cotton

**FIGURE 3:**
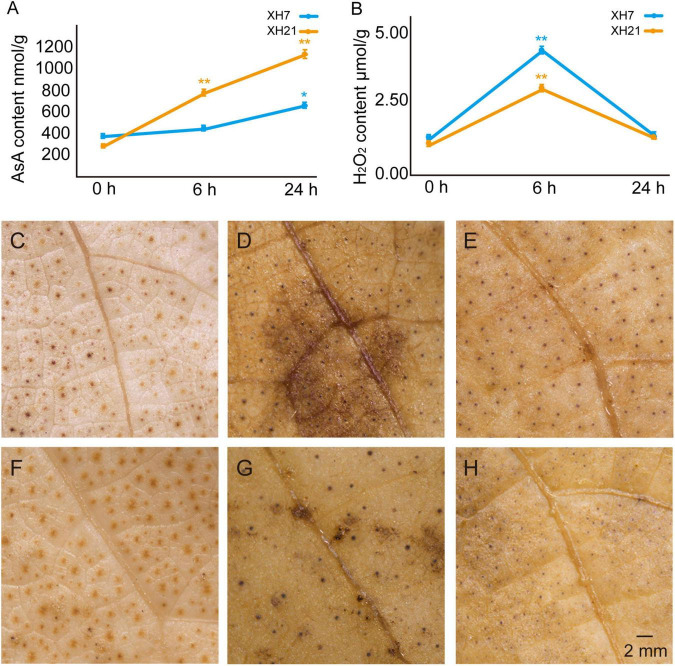
Detection of the AsA and H_2_O_2_ contents in XH7 and XH21 after *V. dahliae* incubation. AsA **(A)** and H_2_O_2_ **(B)** contents were determined in XH7 and XH21 roots treated with *V. dahliae* for 0, 6, and 24 h, respectively. *p < 0.05; **p < 0.01. DAB staining of leaves of XH7 **(C–E)** and XH21 **(F–H)** after *V. dahliae* treatment for 0 h **(C,F)**, 6 h **(D,G)**, and 24 h **(E,H)** are shown. The stained H_2_O_2_ is indicated with a brown color. Bar = 2 mm.

To further confirm the influence of AsA and H_2_O_2_ on the susceptibility of different *G. barbadense* cultivars, we examined the contents of AsA and H_2_O_2_ in the roots of XH7 and XH21 at 0, 6, and 24 h post-infection. The AsA contents of both cultivars were similar before inoculation with *V. dahliae*; however, the AsA contents in XH21 were significantly higher than those in XH7 after treatment with *V. dahliae* (*p* < 0.01, Figure 3A). Correspondingly, the contents of H_2_O_2_ increased shortly after infection (6 h) but then decreased at 24 h post-fungal treatment (Figure 3B). The susceptible cultivar XH7 had a significantly higher (*p* < 0.01) H_2_O_2_ content than that of XH21 at 6 h, and the result was consistent with the lower level of AsA in XH7 at 6 h (Figures 3A,B). To visualize the H_2_O_2_ distribution, DAB staining was performed in cotton leaves from XH7 and XH21 at 0, 6, and 24 h after inoculation with *V. dahliae*. High levels of H_2_O_2_ predominantly accumulated at 6 h in both XH7 and XH21, with a stronger staining signal (dark brown) in XH7 (Figures 3D,G). Exogenous application of AsA onto XH7 and XH21 leaves significantly improved the disease resistance of cotton plants, indicating that extracellular ROS scavenging by peroxidases might be crucial for *V. dahliae* resistance in *G. barbadense* (Supplementary Figure 10).

**FIGURE 4:**
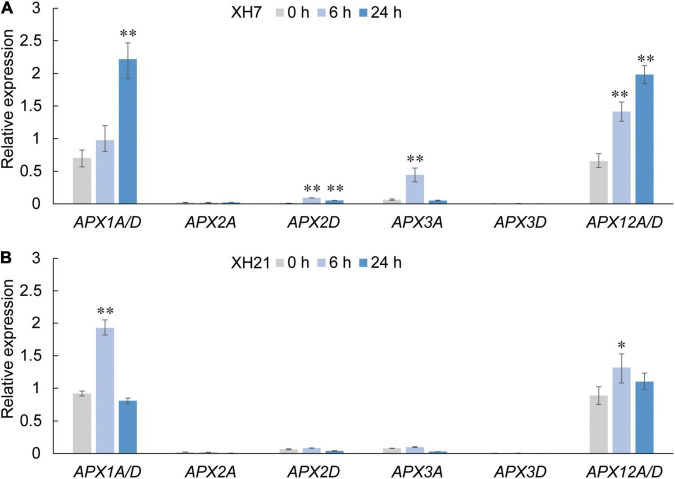
Relative expression levels of *APX* family members in Pima cotton roots of XH7 **(A)** and XH21 **(B)** at 0, 6, and 24 h after *V. dahliae* incubation. Genes with extremely high nucleotide similarity that could not be distinguished by primers were detected using identical primers (*APX1A/D* and *APX12A/D*). *GbUBQ* was used as a reference gene and set to 1. Three independent replicates were performed for each qPCR assay. Significance was analyzed using one-Way ANOVA. *p < 0.05; ^**^p < 0.01.

Thus, we further investigated the mRNA expression levels of *APX* genes, which are considered the only enzymes that catalyze the reduction of H_2_O_2_ using AsA as a specific electron donor. Based on our previous work (Tao et al., 2018) and a transcriptome analysis of *G. barbadense* at different times after *V. dahliae* infection (Supplementary Figure 11, NCBI accession number: PRJNA234454), eight homologs of the *GbAPX* family that were predominantly expressed were selected for qPCR assays (*GbAPX1A/D*, *GbAPX2A/D*, *GbAPX3A/D*, and *GbAPX12A/D*). The results showed that the mRNA levels of partial *GbAPX* homologs were slightly increased in XH21 (no more than twofold change), while most *APX* homologs that were tested here exhibited significantly up-regulated expression levels in XH7, especially *GbAPX1A/D* and *GbAPX12A/D* (Figure 4).

### Silencing *GbAPX1* and *GbAPX12* Compromises the Resistance of Cotton to *Verticillium dahliae*

**FIGURE 5:**
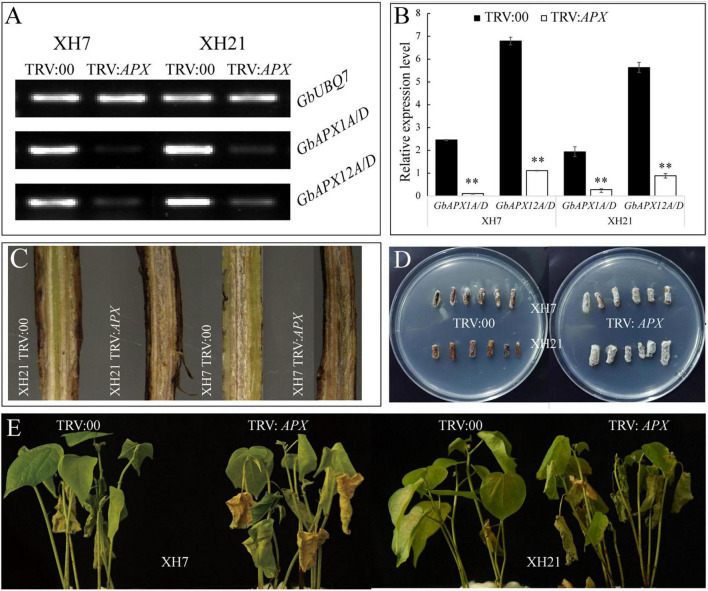
The resistance of VIGS plants to *V. dahlia* was compromised. Semi-quantitative **(A)** and real-time quantitative **(B)** PCR were used to select successfully silenced transformants. Three replicates were performed for each transformed plant. ^**^p < 0.01. **(C)** Fungal accumulation in the stems of TRV:*00* and TRV:*GbAPX1/12* plants. **(D)** Fungal hyphae recovery assays of the *V. dahlia*-infected cotton. The stem sections were plated on PDA medium, incubated at 25°C, and photographed at 5 days post-plating. **(E)** Disease symptoms for the representative plants of TRV:00 and TRV:*GbAPX1/12* at 14 days after inoculation. *N* = 18 for each treatment.

To validate the functions of the predominant *GbAPX* members in *V. dahliae* resistance, conserved fragments of *GbAPX1A/D* and *GbAPX12A/D* were used to construct a VIGS vector (TRV:*GbAPX1/12*). Successful silencing of the positive control and target genes was confirmed by semi and real-time quantitative PCR (Supplementary Figure 12 and Figures 5A,B). The *V. dahliae* accumulation in the stem of *GbAPX1/12*-silenced transformants was more severe than that in the TRV:00 control at 14 days after *V. dahliae* inoculation in both XH7 and XH21, and more dark brown streaks were observed in the stems (Figure 5C). In the fungal recovery assays, more hyphae around stem sections were observed with the *GbAPX1/12*-silenced plants than with the TRV:00 controls (Figure 5D). As a result, the disease symptoms observed for the TRV:00 plants were similar to those of regular wild type plants (XH7 is susceptible and XH21 is resistant), while TRV:*GbAPX1/12* plants of both XH7 and XH21 showed similar disease symptoms, and these symptoms were much more severe than those of TRV:00 (Figure 5E). Together, silencing predominantly expressed APX family members in Pima cotton compromises the resistance to *V. dahliae.*

## Discussion

In total, 885 DAPs were identified at 0, 6, and 24 h after infection in XH7 and XH21, and a much higher number of DAPs were observed than those identified in 2-DE based studies (Witzel et al., 2017). Benefiting from the high sensitivity, many novel DAPs have been identified, such as low-abundant transcript factors (nuclear transport factor 2 -like protein, transcription factor RF2a, GATA transcription factor 26 -like protein) and very small molecular weight peptides (malate dehydrogenase-2C mitochondrial, cytochrome b-c1 complex subunit 9), which are very difficult to be detected by 2-DE based proteomic techniques (Supplementary Table 2). The WGCNA and pathway enrichment analyses of the module with the highest module-trait relationship revealed the key pathways that are involved in VW resistance in *G. barbadense* (Supplementary Figures 4, 5). Some of the enriched pathways were mentioned in previous works, such as the response to oxidative stress (Hu et al., 2019). In addition to the common DAPs, DAPs that are specific to the resistant cotton XH21 also represented biological significance for cotton VW resistance. Both pathway enrichment analyses of common and XH21-specific DAPs revealed that ROS-related pathways, especially the biological processes related to H_2_O_2_ scavenging, were significantly enriched (Supplementary Figures 5, 6). Ribosomal protein GaRPL18 contributes significantly to cotton resistance (Gong et al., 2017). In this study, ribosomal-related pathways were also observed to be significantly enriched pathways and can be studied for the function of these proteins in cotton disease resistance in future investigations.

Ascorbate acid has been demonstrated to play various important roles in cotton, including fiber development and stress response (Ma et al., 2019; Pan et al., 2019; Song et al., 2019). It is well known that the antioxidant system is important for improving plant resistance to abiotic or biotic stress; however, few studies have reported the functions of AsA in *V. dahliae* resistance in *G. barbadense*. Here, we examined the AsA and H_2_O_2_ contents in resistant and susceptible cultivars, showing that higher AsA contents and lower H_2_O_2_ levels were closely correlated with the disease resistance (Figure 3). The different levels of AsA and H_2_O_2_ between high- and low-resistance *G. barbadens* cultivars could be partly explained by our data for the XH21-speicific accumulated protein *GLDH* (Supplementary Figure 9), which is responsible for AsA biosynthesis, and by the higher expression levels of the class I peroxidase *APX* in XH7 (Figure 4). Exogenous application of AsA onto XH7 and XH21 plants significantly improved their VW syptoms (Supplementary Figure 10).

Ascorbate peroxidase are necessary for cotton fiber development (Li et al., 2007; Guo et al., 2016); however, thus far, no study has linked APX to pathogen resistance in cotton species, although several investigations have shown that APX activity is important for the tolerance of rice and wheat to pathogens (Gou et al., 2015; Jiang et al., 2016). Our data showed that APXs might be related to cotton VW resistance by regulating redox homeostasis. The qPCR of *APXs*, coupled with the AsA and H_2_O_2_ content assays, might provide a possible explanation for the high *V. dahliae* resistance of XH21, which was mainly attributed to the high activity of AsA biosynthesis and the high levels of AsA in XH21. In contrast, the AsA levels in XH7 were much lower than those in XH21, possibly because of the low level of *GbGLDH* and significantly increased expression of *GbAPX*, which consumes AsA as an electron donor. This was further confirmed by gene silencing experiments. TRV:00 transformants exhibited disease symptoms similar to those of their original phenotypes; cultivar XH7 was susceptible and XH21 was resistant. However, by knocking down *GbAPX1/12* expression, the transformants of TRV:*GbAPX1/12* exhibited a significantly decreased resistance to *V. dahliae* in both XH7 and XH21 (Figure 5E).

## Conclusion

In summary, we identified many novel DAPs by using a DIA-based high-throughput proteomic analysis in two *G. barbadense* varieties with different VW resistance. WGCNA and pathway enrichment analyses revealed the key pathways that are involved in VW resistance in *G. barbadense.* Increased AsA level, decreased H_2_O_2_ content, were observed in VW resistant variety XH21. Knocking down *GbAPX1/12* expression in *G. barbadense* resulted in significantly decreased resistance to *V. dahlia* in both XH7 and XH21. Our results provide effective proteomic references for elucidating the VW resistance mechanism and genetic improvement of VW resistant cotton germplasms.

## Data Availability Statement

The datasets presented in this study can be found in online repository ProteomeXchange Consortium (http://proteomecentral.proteomexchange.org) under the identifier PXD017527.

## Author Contributions

RL, XJ, and HL contributed to conception and design of the study. TL, LZ, YL, FW, AC, SX, XC, HS, BW, and MH performed the experiment and data analyses. TL and LZ performed the statistical analysis. TL and XJ wrote the first draft of the manuscript. LZ, RL, XJ, and HL wrote sections of the manuscript. All authors contributed to manuscript revision, read, and approved the submitted version.

## Conflict of Interest

The authors declare that the research was conducted in the absence of any commercial or financial relationships that could be construed as a potential conflict of interest.

## Publisher’s Note

All claims expressed in this article are solely those of the authors and do not necessarily represent those of their affiliated organizations, or those of the publisher, the editors and the reviewers. Any product that may be evaluated in this article, or claim that may be made by its manufacturer, is not guaranteed or endorsed by the publisher.

## Footnotes

1
http://www.heatmapper.ca/expression/

## Supplementary Material

Supplementary Figure 1 Disease index of XH7 and XH21 after *V. dahliae* incubation. The number of four represented the highest disease index when the whole plant died, and the number of zero indicated the lowest disease index with no visible wilting. The numbers zero to four are also presented by different colors for visualization.

Supplementary Figure 2 Venn diagram for three replicates of proteomics data. The diagram shows the distribution of the identified proteins in three independent experiments, indicating a high repetitiveness of three replicates in each group.

Supplementary Figure 3 Statistical analysis of DAPs. Venn diagram of DAPs at 6 and 24 h compared to 0 h in XH7 **(A)** and XH21 **(D)**. The red and green numbers indicate up- and down-regulated protein respectively. Volcano plots of DAPs at 6 h compared to 0 h in XH7 **(B)** and XH21 **(E)**; volcano plots of DAPs of 24 h compared to 0 h in XH7 **(C)** and XH21 **(F)**; the red and green dots represented increased and decreased abundant proteins, respectively, and the blue dots represent proteins without significance.

Supplementary Figure 4 Weighted gene co-expression network analysis of 885 DAPs. **(A)** Network heatmap of DAPs. Recognized modules are indicated by different colors. The light color represents a low overlap, and darker red indicates a higher overlap between proteins. **(B)** Module-trait relationship of eight well-coexpression modules. The depth of color corresponds to the correlation. Positive correlations are indicated in red color, and negative correlations are represented in blue color. Significance (*p*-value) of each module to time or variety presented in parentheses.

Supplementary Figure 5 GO enrichment of the 255 DAPs from the turquoise module, which showed the most significant positive relationships with infection time. **(A)** Biological Process. **(B)** Cellular Component. **(C)** Molecular Function. The sphere size indicates the number of genes in the corresponding term; the color corresponds to different correct *p*-value ranges. Gray lines connect the terms with related functional enrichment.

Supplementary Figure 6 Tree-view of GO terms for the molecular function category enriched by 184 XH21-specific DAPs. The depth of color corresponds to the *p* value of significance. The GO ID, *p*-value, annotation and the numbers of DAPs for each GO term are shown inside the boxes. The GO ID with red color font indicates the subsequent experimental verification term selected in this study.

Supplementary Figure 7 Tree-view of GO terms for the biological process category enriched by the 184 XH21-specific DAPs. The white box represents GO category terms. The depth of color corresponds to the statistical significance. The GO ID, *p*-value, annotation and the numbers of DAPs for each GO term are shown inside the boxes. Stars indicate that the terms are not on the key nodes of the tree.

Supplementary Figure 8 Tree-view of GO terms for the cellular component category enriched by the 184 XH21-specific DAPs. The white box represents GO category terms. The depth of color corresponds to the statistical significance. The GO ID, *p*-value, annotation and the numbers of DAPs for each GO term are shown inside the boxes. Stars indicate that the terms are not on the key nodes of the tree.

Supplementary Figure 9 qRT–PCR validation of the expression levels of the nine proteins in GO:0050662, coenzyme binding. The transcriptional expression levels of the nine proteins distributed in the coenzyme binding category of GO:0050662, including flavin-containing monooxygenase YUCCA10-like protein (*YUCCA10*), peroxisomal acyl-coenzyme A oxidase 1-like protein (*Acox1*), FAD/NAD(P)-binding oxidoreductase family protein isoform 1 (*FAD*), dihydroflavonol-4-reductase (*DFR*), thiamine pyrophosphate dependent pyruvate decarboxylase family protein (*PDC*), bifunctional aspartokinase (*AKHSD*), thiamine pyrophosphate dependent pyruvate decarboxylase family protein (*PDC2*), glucose-6-phosphate 1-dehydrogenase-2C chloroplastic (*G6PD1*), and l-galactono-1,4-lactone dehydrogenase-2C mitochondrial-like protein (*GLDH*), were measured by qRT–PCR with relative expression levels to the reference gene of *UBQ*. Finally, the fold change between the infected groups and the control is shown, and the expression level in the 0 h samples is set to 1. Three independent replicates were performed for each qPCR assay. **p* < 0.05; ^**^*p* < 0.01.

Supplementary Figure 10 Disease index of XH7 and XH21 with or without exogenous AsA application after *V. dahliae* treatment. The exogenous applications of 0.5 mM AsA were performed when the cotton seedlings were exposed to *V. dahliae*, and H_2_O was used as a control. The fungal treatment time was extended to 3 days. Then, the seedlings were transferred into Hoagland’s nutrient solution to measure the disease index. The number four was the highest disease index when the whole plant died, and zero indicated the lowest disease index with no visible wilting. The numbers zero to four are also illustrated by different colors for visualization.

Supplementary Figure 11 Heatmap of transcriptional expression levels of all *G. barbadense APX* genes deduced from public transcriptome data of *V. dahlia*-treated roots. The transcriptome data of *G. barbadense* roots after *V. dahliae* treatment were obtained from the public online database at NCBI (accession number: PRJNA234454). The colors of yellow, black, and blue indicate high, moderate, and low transcriptional expression levels, respectively. The heatmap was produced by the value of Log_2_ of FPKM by Heatmapper (http://www.heatmapper.ca/expression/). The *APX* genes of red fonts were selected for further analysis of qRT–PCR validation.

Supplementary Figure 12 Phenotypes and qPCR assays of the positive control. TRV:*GbCLA* was used as a positive control for VIGS. Silencing of *GbCLA* will prevent the biosynthesis of chlorophyll and result in write leaves [**(A)** for XH7 and **(B)** for XH21]. qPCR of *GbCLA* showed a significant decrease of expression level in transformed plants **(C)**.

Supplementary Table 1 Primers used in this work.

Supplementary Table 2 Detailed information for the 885 DAPs.

Supplementary Table 3 Detailed information for the 184 XH21-specific DAPs.

## References

[B1] Babicki S. Arndt D. Marcu A. Liang Y. Grant J. R. Maciejewski A. (2016). Heatmapper: web-enabled heat mapping for all. *Nucleic Acids Res.* 44 147–153. 10.1093/nar/gkw419 27190236 PMC4987948

[B2] Bernhardt O. M. Selevsek N. Gillet L. C. Rinner O. Picotti P. Aebersold R. (2012). “Spectronaut: a fast and efficient algorithm for MRM-like processing of data independent acquisition (SWATH-MS) data,” in *Proceedings of the 60th ASMS Conference on Mass Spectrometry and Allied Topics*, Vancouver.

[B3] Bradford M. M. (1976). A rapid and sensitive method for the quantitation of microgram quantities of protein utilizing the principle of protein–dye binding. *Anal. Biochem.* 72 248–254. 10.1006/abio.1976.9999 942051

[B4] Bruderer R. Bernhardt O. M. Gandhi T. Xuan Y. Sondermann J. Schmidt M. (2017). Optimization of experimental parameters in data-independent mass spectrometry significantly increases depth and reproducibility of results. *Mol. Cell. Proteomics* 16 2296–2309. 10.1074/mcp.RA117.000314 29070702 PMC5724188

[B5] Burch-Smith T. M. Anderson J. C. Martin G. B. Dinesh-Kumar S. P. (2004). Applications and advantages of virus-induced gene silencing for gene function studies in plants. *Plant J.* 39 734–746. 10.1111/j.1365-313X.2004.02158.x 15315635

[B6] Cheng H. Q. Han L. B. Yang C. L. Wu X. M. Zhong N. Q. Wu J. H. (2016). The cotton MYB108 forms a positive feedback regulation loop with CML11 and participates in the defense response against *Verticillium dahliae* infection. *J. Exp. Bot.* 67 1935–1950. 10.1093/jxb/erw016 26873979 PMC4783372

[B7] Escher C. Reiter L. MacLean B. Ossola R. Herzog F. Chilton J. (2012). Using iRT, a normalized retention time for more targeted measurement of peptides. *Proteomics* 12 1111–1121. 10.1002/pmic.201100463 22577012 PMC3918884

[B8] Fang W. Xie D. Zhu H. Li W. Xu Z. Yang L. (2015). Comparative proteomic analysis of *Gossypium thurberi* in response to *Verticillium dahliae* inoculation. *Int. J. Mol. Sci.* 16 25121–25140. 10.3390/ijms161025121 26506344 PMC4632794

[B9] Gabriela B. Bernhard M. Hubert H. Pornpimol C. Marie T. Amos K. (2009). Cluego: a cytoscape plug-in to decipher functionally grouped gene ontology and pathway annotation networks. *Bioinformatics* 8 1091–1093. 10.1093/bioinformatics/btp101 19237447 PMC2666812

[B10] Gong Q. Yang Z. Wang X. Butt H. I. Chen E. He S. (2017). Salicylic acid-related cotton (*Gossypium arboreum*) ribosomal protein *GaRPL18* contributes to resistance to *Verticillium dahliae*. *BMC Plant Biol.* 17:59. 10.1186/s12870-017-1007-5 28253842 PMC5335750

[B11] Gou J. Y. Li K. Wu K. Wang X. Lin H. Cantu D. (2015). Wheat stripe rust resistance protein wks1 reduces the ability of the thylakoid-associated ascorbate peroxidase to detoxify reactive oxygen species. *Plant Cell* 27 1755–1770. 10.1105/tpc.114.134296 25991734 PMC4498197

[B12] Guo K. Du X. Tu L. Tang W. Wang P. Wang M. (2016). Fibre elongation requires normal redox homeostasis modulated by cytosolic ascorbate peroxidase in cotton (*Gossypium hirsutum*). *J. Exp. Bot.* 67 3289–3301. 10.1093/jxb/erw146 27091877 PMC4892722

[B13] Han L. B. Li Y. B. Wang F. X. Wang W. Y. Liu J. Wu J. H. (2019). The cotton apoplastic protein CRR1 stabilizes chitinase 28 to facilitate defense against the fungal pathogen *Verticillium dahliae*. *Plant Cell* 31 520–536. 10.1105/tpc.18.00390 30651348 PMC6447012

[B14] Hiraga S. Sasaki K. Ito H. Ohashi Y. Matsui H. (2001). A large family of class III plant peroxidases. *Plant Cell Physiol.* 42 462–468. 10.1093/pcp/pce061 11382811

[B15] Hoagland D. R. (1920). Optimum nutrient solutions for plants. *Science* 52 562–564. 10.1126/science.52.1354.562 17811355

[B16] Hu X. Puri K. D. Gurung S. Klosterman S. J. Wallis C. M. Britton M. (2019). Proteome and metabolome analyses reveal differential responses in tomato -*Verticillium dahliae*-interactions. *J. Proteomics* 207:103449. 10.1016/j.jprot.2019.103449 31323424

[B17] Jiang G. Yin D. Zhao J. Chen H. Guo L. Zhu L. (2016). The rice thylakoid membrane-bound ascorbate peroxidase *OsAPX8* functions in tolerance to bacterial blight. *Sci. Rep.* 6:26104. 10.1038/srep26104 27185545 PMC4868969

[B18] Jin X. Zhu L. Tao C. Xie Q. Xu X. Chang L. (2019). An improved protein extraction method applied to cotton leaves is compatible with 2-DE and LC-MS. *BMC Genomics* 20:285. 10.1186/s12864-019-5658-5 30975097 PMC6458646

[B19] Klosterman S. J. Atallah Z. K. Vallad G. E. Subbarao K. V. (2009). Diversity, pathogenicity, and management of *Verticillium* species. *Annu. Rev. Phytopathol.* 47 39–62. 10.1146/annurev-phyto-080508-081748 19385730

[B20] Langfelder P. Horvath S. (2008). WGCNA: an R package for weighted correlation network analysis. *BMC Bioinformatics* 9:559. 10.1186/1471-2105-9-559 19114008 PMC2631488

[B21] Li H. B. Qin Y. M. Pang Y. Song W. Q. Mei W. Q. Zhu Y. X. (2007). A cotton ascorbate peroxidase is involved in hydrogen peroxide homeostasis during fibre cell development. *New Phytol.* 175 462–471. 10.1111/j.1469-8137.2007.02120.x 17635221

[B22] Li N. Y. Zhou L. Zhang D. D. Klosterman S. J. Li T. G. Gui Y. J. (2018). Heterologous expression of the cotton NBS-LRR gene *GbaNA1* enhances *Verticillium* wilt resistance in *Arabidopsis*. *Front. Plant Sci.* 9:119. 10.3389/fpls.2018.00119 29467784 PMC5808209

[B23] Li Y. B. Han L. B. Wang H. Y. Zhang J. Sun S. T. Feng D. Q. (2016). The thioredoxin *GbNRX1* plays a crucial role in homeostasis of apoplastic reactive oxygen species in response to *Verticillium dahliae* infection in cotton. *Plant Physiol.* 170 2392–2406. 10.1104/pp.15.01930 26869704 PMC4825149

[B24] Li Z. K. Chen B. Li X. X. Wang J. P. Zhang Y. Wang X. F. (2019). A newly identified cluster of glutathione S-transferase genes provides *Verticillium* wilt resistance in cotton. *Plant J.* 98 213–227. 10.1111/tpj.14206 30561788

[B25] Liu N. Sun Y. Wang P. Duan H. Ge X. Li X. (2018b). Mutation of key amino acids in the polygalacturonase-inhibiting proteins *CkPGIP1* and *GhPGIP1* improves resistance to *Verticillium* wilt in cotton. *Plant J.* 96 546–561. 10.1111/tpj.14048 30053316

[B26] Liu N. Sun Y. Pei Y. Zhang X. Wang P. Li X. (2018a). A pectin methylesterase inhibitor enhances resistance to *Verticillium* wilt. *Plant Physiol.* 176 2202–2220. 10.1104/pp.17.01399 29363564 PMC5841709

[B27] Ma R. Song W. Wang F. Cao A. Xie S. Chen X. (2019). A cotton (*Gossypium hirsutum*) *Myo*-inositol-1-phosphate synthase (*GhMIPS1D*) gene promotes root cell elongation in *Arabidopsis*. *Int. J. Mol. Sci.* 20:1224. 10.3390/ijms20051224 30862084 PMC6429088

[B28] Ma Z. Wang X. Zhang G. Liu S. Sun J. Liu J. (1999). Genetic studies of *Verticillium* wilt resistance among different types of sea island cottons. *Zuo Wu Xue Bao* 26 321–345.

[B29] Mellidou I. Kanellis A. K. (2017). Genetic control of ascorbic acid biosynthesis and recycling in horticultural crops. *Front. Chem.* 5:50. 10.3389/fchem.2017.00050 28744455 PMC5504230

[B30] Mittler R. Vanderauwera S. Gollery M. Van Breusegem F. (2004). Reactive oxygen gene network of plants. *Trends Plant Sci.* 9 490–498. 10.1016/j.tplants.2004.08.009 15465684

[B31] Okazaki Y. Ishizuka A. Ishihara A. Nishioka T. Iwamura H. (2007). New dimeric compounds of avenanthramide phytoalexin in oats. *J. Org. Chem.* 72 3830–3839. 10.1021/jo0701740 17432911

[B32] Pan Z. Chen L. Wang F. Song W. Cao A. Xie S. (2019). Genome-wide identification and expression analysis of the ascorbate oxidase gene family in *Gossypium hirsutum* reveals the critical role of *GhAO1A* in delaying dark-induced leaf senescence. *Int. J. Mol. Sci.* 20:6167. 10.3390/ijms20246167 31817730 PMC6940856

[B33] Passardi F. Penel C. Dunand C. (2004). Performing the paradoxical: how plant peroxidases modify the cell wall. *Trends Plant Sci.* 9 534–540. 10.1016/j.tplants.2004.09.002 15501178

[B34] Pino L. K. Just S. C. MacCoss M. J. Searle B. C. (2020). Acquiring and analyzing data independent acquisition proteomics experiments without spectrum libraries. *Mol. Cell. Proteomics* 19 1088–1103. 10.1074/mcp.P119.001913 32312845 PMC7338082

[B35] Roe M. R. Griffin T. J. (2006). Gel-free mass spectrometry-based high throughput proteomics: tools for studying biological response of proteins and proteomes. *Proteomics* 6 4678–4687. 10.1002/pmic.200500876 16888762

[B36] Shaban M. Miao Y. Ullah A. Khan A. Q. Menghwar H. Khan A. H. (2018). Physiological and molecular mechanism of defense in cotton against *Verticillium dahliae*. *Plant Physiol. Biochem.* 125 193–204. 10.1016/j.plaphy.2018.02.011 29462745

[B37] Smirnoff N. Arnaud D. (2019). Hydrogen peroxide metabolism and functions in plants. *New Phytol.* 221 1197–1214. 10.1111/nph.15488 30222198

[B38] Song R. Li J. Xie C. Jian W. Yang X. (2020). An overview of the molecular genetics of plant resistance to the *Verticillium* wilt pathogen *Verticillium dahliae*. *Int. J. Mol. Sci.* 21:E1120. 10.3390/ijms21031120 PMC7037454 32046212

[B39] Song W. Wang F. Chen L. Ma R. Zuo X. Cao A. (2019). *GhVTC1*, the key gene for ascorbate biosynthesis in *Gossypium hirsutum*, involves in cell elongation under control of ethylene. *Cells* 8:1039. 10.3390/cells8091039 31492030 PMC6769745

[B40] Tao C. Jin X. Zhu L. Xie Q. Wang X. Li H. (2018). Genome-wide investigation and expression profiling of *APX* gene family in *Gossypium hirsutum* provide new insights in redox homeostasis maintenance during different fiber development stages. *Mol. Genet. Genomics* 293 685–697. 10.1007/s00438-017-1413-2 29307114 PMC5948307

[B41] Tian T. Liu Y. Yan H. You Q. Yi X. Du Z. (2017). agriGO v2.0: a GO analysis toolkit for the agricultural community, 2017 update. *Nucleic Acids Res.* 45 122–129. 10.1093/nar/gkx382 28472432 PMC5793732

[B42] Wang F. X. Luo Y. M. Ye Z. Q. Cao X. Liang J. N. Wang Q. (2018). iTRAQ-based proteomics analysis of autophagy-mediated immune responses against the vascular fungal pathogen *Verticillium dahliae* in *Arabidopsis*. *Autophagy* 14 598–618. 10.1080/15548627.2017.1423438 29369001 PMC5959329

[B43] Wang M. Tu L. Yuan D. Zhu D. Shen C. Li J. (2019). Reference genome sequences of two cultivated allotetraploid cottons, *Gossypium hirsutum* and *Gossypium barbadense*. *Nat. Genet.* 51 224–229. 10.1038/s41588-018-0282-x 30510239

[B44] Witzel K. Buhtz A. Grosch R. (2017). Temporal impact of the vascular wilt pathogen *Verticillium dahliae* on tomato root proteome. *J. Proteomics* 169 215–224. 10.1016/j.jprot.2017.04.008 28428141

[B45] Wu L. B. Ueda Y. Lai S. K. Frei M. (2017). Shoot tolerance mechanisms to iron toxicity in rice (*Oryza sativa* L.). *Plant Cell Environ.* 40 570–584. 10.1111/pce.12733 26991510

[B46] Wu X. Yan J. Wu Y. Zhang H. Mo S. Xu X. (2019). Proteomic analysis by iTRAQ-PRM provides integrated insight into mechanisms of resistance in pepper to *Bemisia tabaci* (Gennadius). *BMC Plant Biol.* 19:270. 10.1186/s12870-019-1849-0 31226939 PMC6588876

[B47] Yang J. Wang X. Xie M. Wang G. Li Z. Zhang Y. (2020). Proteomic analyses on xylem sap provides insights into the defense response of *Gossypium hirsutum* against *Verticillium dahliae*. *J. Proteomics* 213:103599. 10.1016/j.jprot.2019.103599 31809902

[B48] Yang J. Zhang Y. Wang X. Wang W. Li Z. Wu J. (2018). *HyPRP1* performs a role in negatively regulating cotton resistance to *V. dahliae* via the thickening of cell walls and ROS accumulation. *BMC Plant Biol.* 18:339. 10.1186/s12870-018-1565-1 30526498 PMC6286592

[B49] Zhang X. Cheng W. Feng Z. Zhu Q. Sun Y. Li Y. (2020). Transcriptomic analysis of gene expression of *Verticillium dahliae* upon treatment of the cotton root exudates. *BMC Genomics* 21:155. 10.1186/s12864-020-6448-9 PMC7017574 32050898

[B50] Zheng Y. Xu J. Wang F. Tang Y. Wei Z. Ji Z. (2021). Mutation types of CYP71P1 cause different phenotypes of mosaic spot lesion and premature leaf senescence in rice. *Front. Plant Sci.* 12:641300. 10.3389/fpls.2021.641300 PMC8021961 33833770

[B51] Zhou Y. Sun L. Wassan G. M. He X. Shaban M. Zhang L. (2019). GbSOBIR1 confers *Verticillium* wilt resistance by phosphorylating the transcriptional factor *GbHLH171* in *Gossypium barbadense*. *Plant Biotechnol. J.* 17 152–163. 10.1111/pbi.12954 29797390 PMC6330551

