# Supplementary figures and images for: Comparative Proteomic Analysis Reveals the Ascorbate Peroxidase-Mediated Plant Resistance to Verticillium dahliae in Gossypium barbadense
Source: Front Plant Sci. 2022 May 19;13:877146. doi: 10.3389/fpls.2022.877146 (PMC9161280; doi:10.3389/fpls.2022.877146)

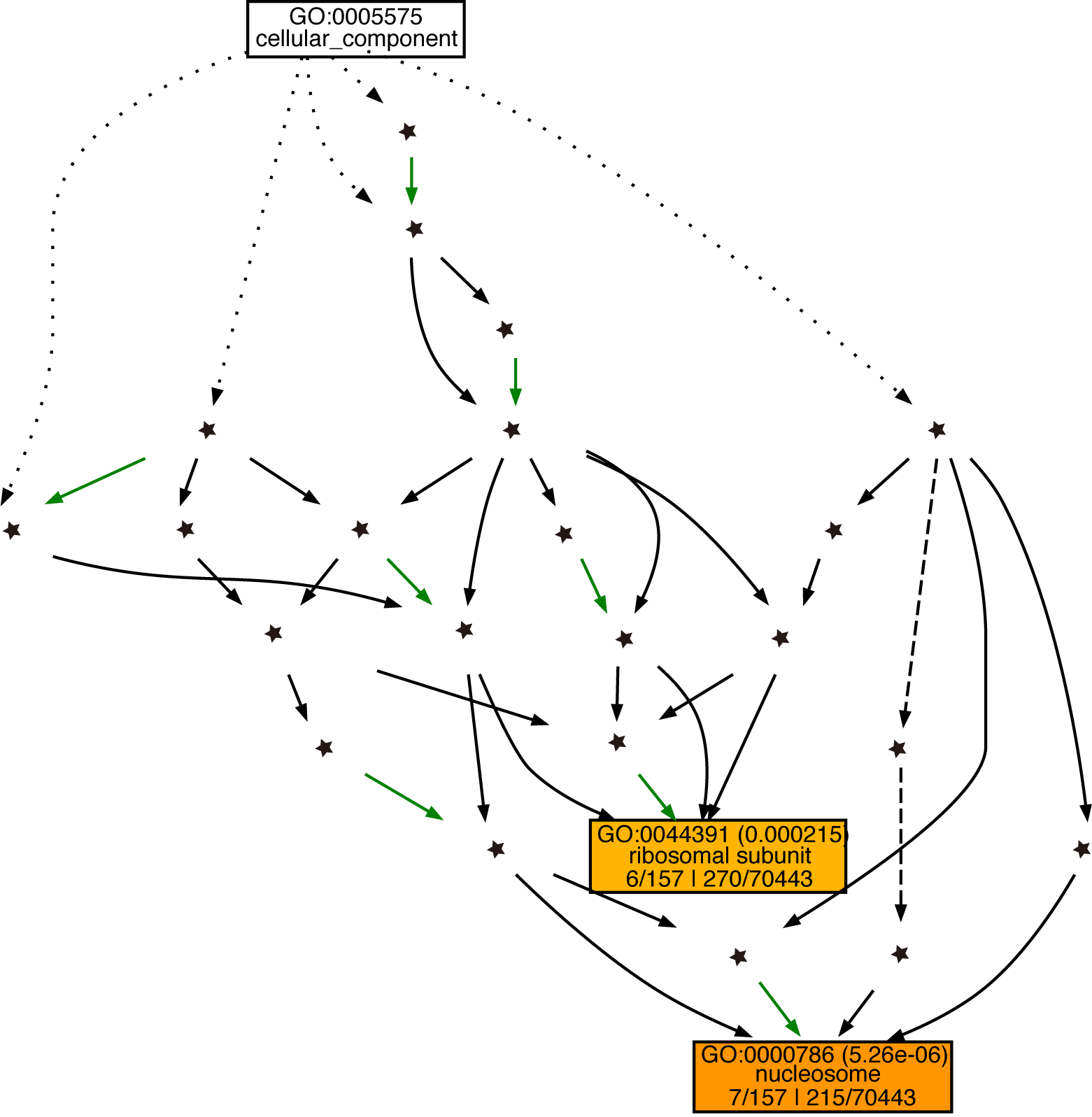

Supplement: Supplementary Figure 1 — Disease index of XH7 and XH21 after V. dahliae incubation. The number of four represented the highest disease index when the whole plant died, and the number of zero indicated the lowest disease index with no visible wilting. The numbers zero to four are also presented by different colors for visualization. [file Data_Sheet_1.zip › sFigures&Tables/Figure S8.tif]

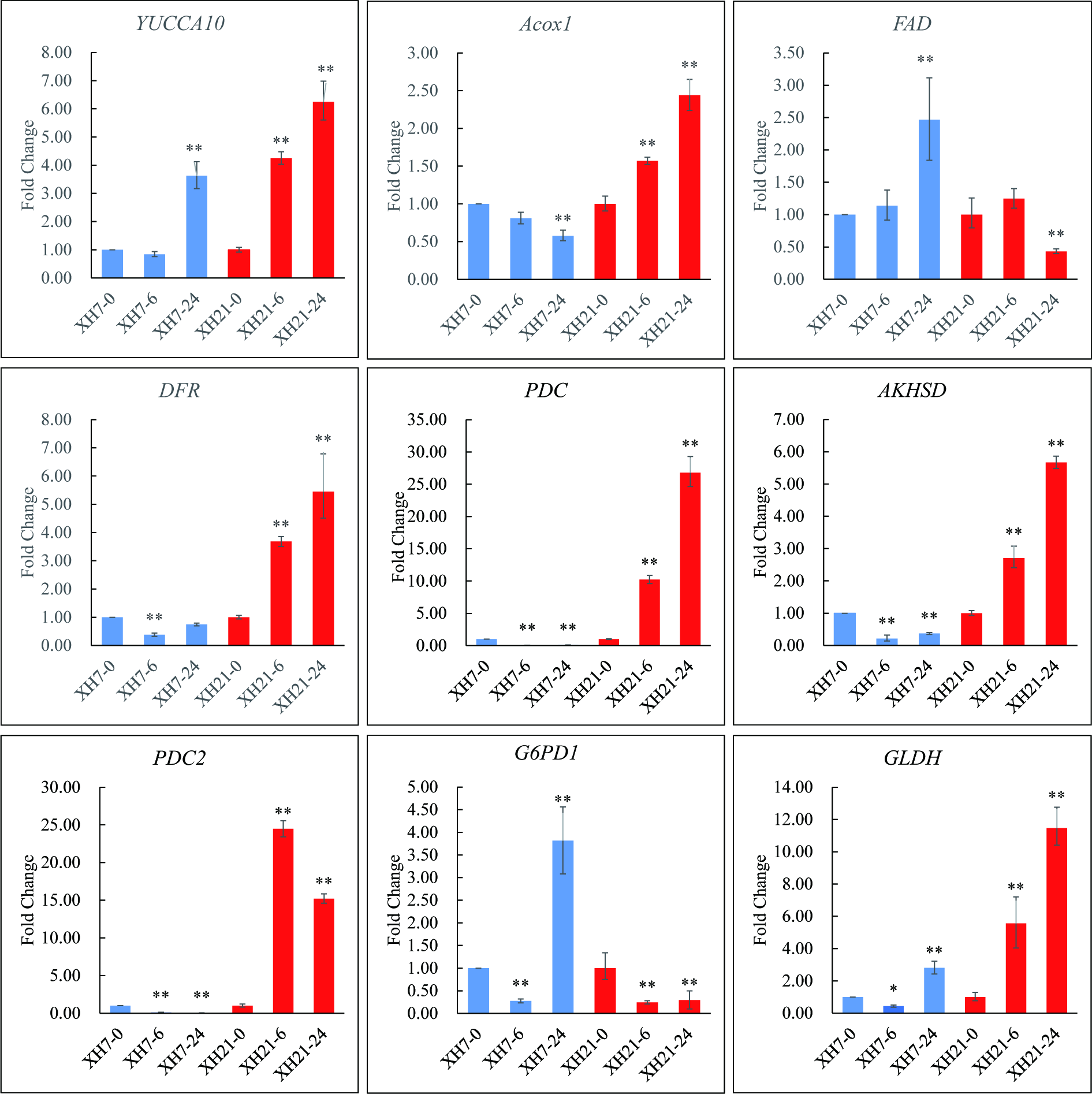

Supplement: Supplementary Figure 1 — Disease index of XH7 and XH21 after V. dahliae incubation. The number of four represented the highest disease index when the whole plant died, and the number of zero indicated the lowest disease index with no visible wilting. The numbers zero to four are also presented by different colors for visualization. [file Data_Sheet_1.zip › sFigures&Tables/Figure S9.tif]

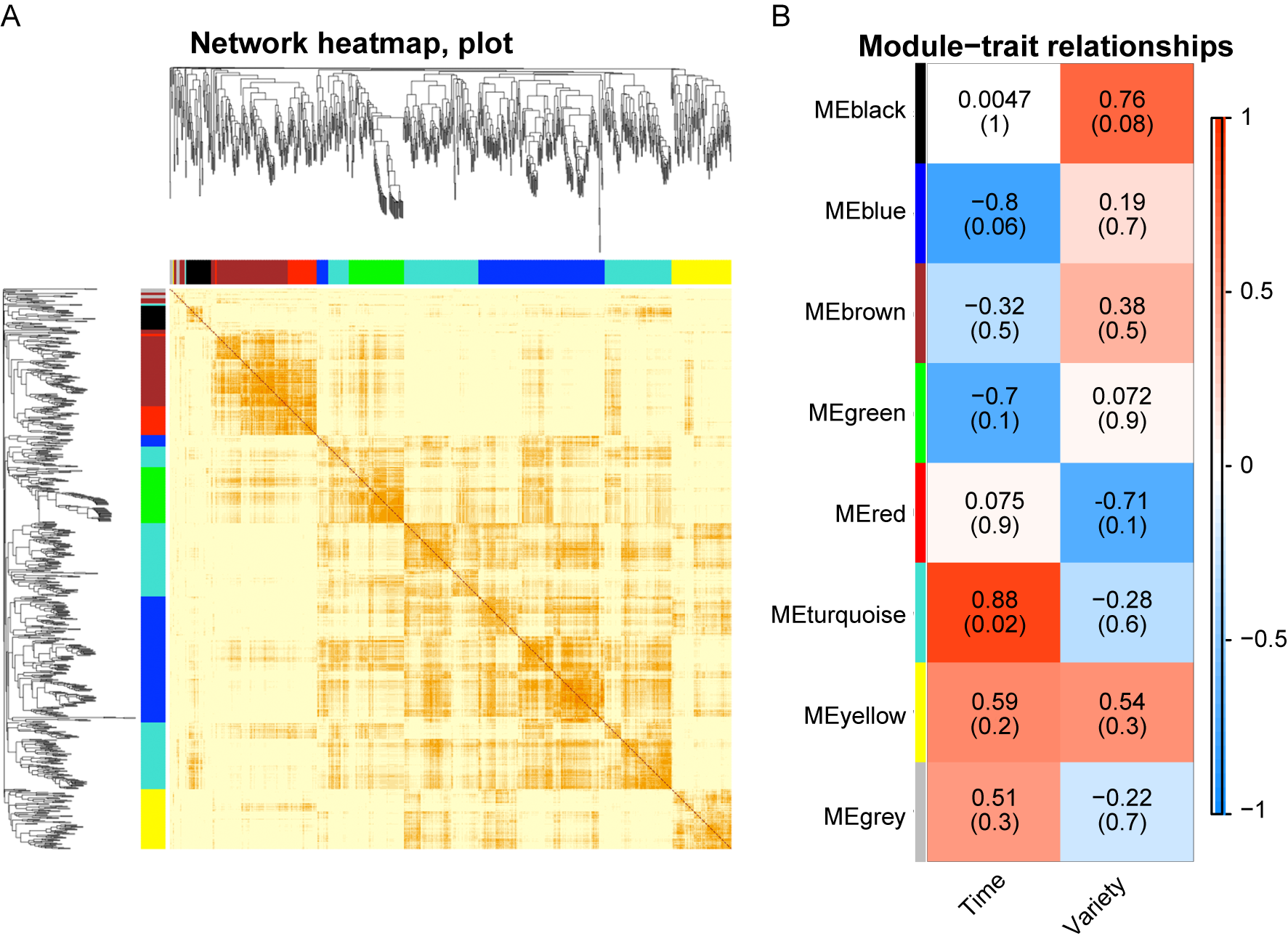

Supplement: Supplementary Figure 1 — Disease index of XH7 and XH21 after V. dahliae incubation. The number of four represented the highest disease index when the whole plant died, and the number of zero indicated the lowest disease index with no visible wilting. The numbers zero to four are also presented by different colors for visualization. [file Data_Sheet_1.zip › sFigures&Tables/Figure S4.tif]

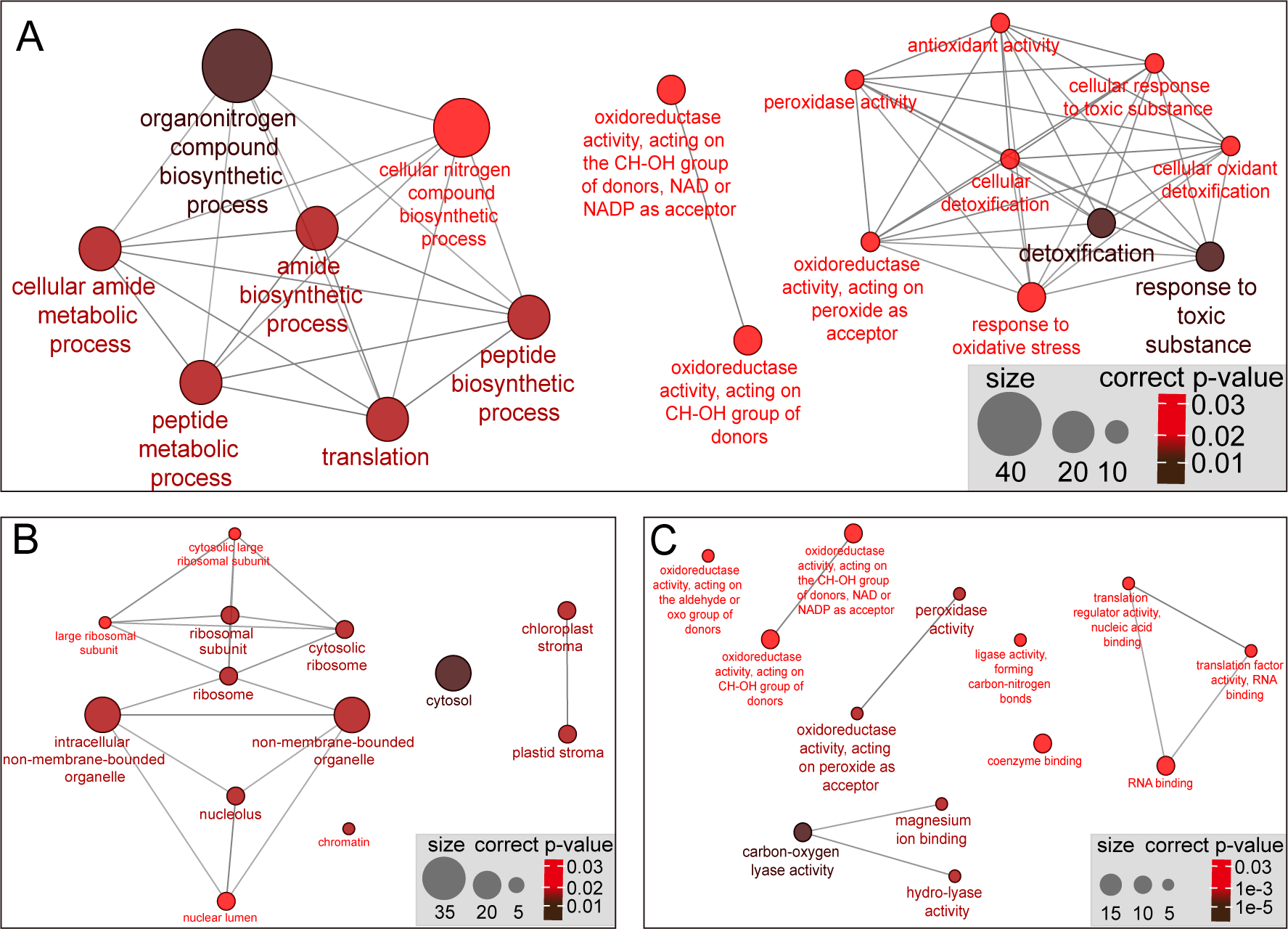

Supplement: Supplementary Figure 1 — Disease index of XH7 and XH21 after V. dahliae incubation. The number of four represented the highest disease index when the whole plant died, and the number of zero indicated the lowest disease index with no visible wilting. The numbers zero to four are also presented by different colors for visualization. [file Data_Sheet_1.zip › sFigures&Tables/Figure S5.tif]

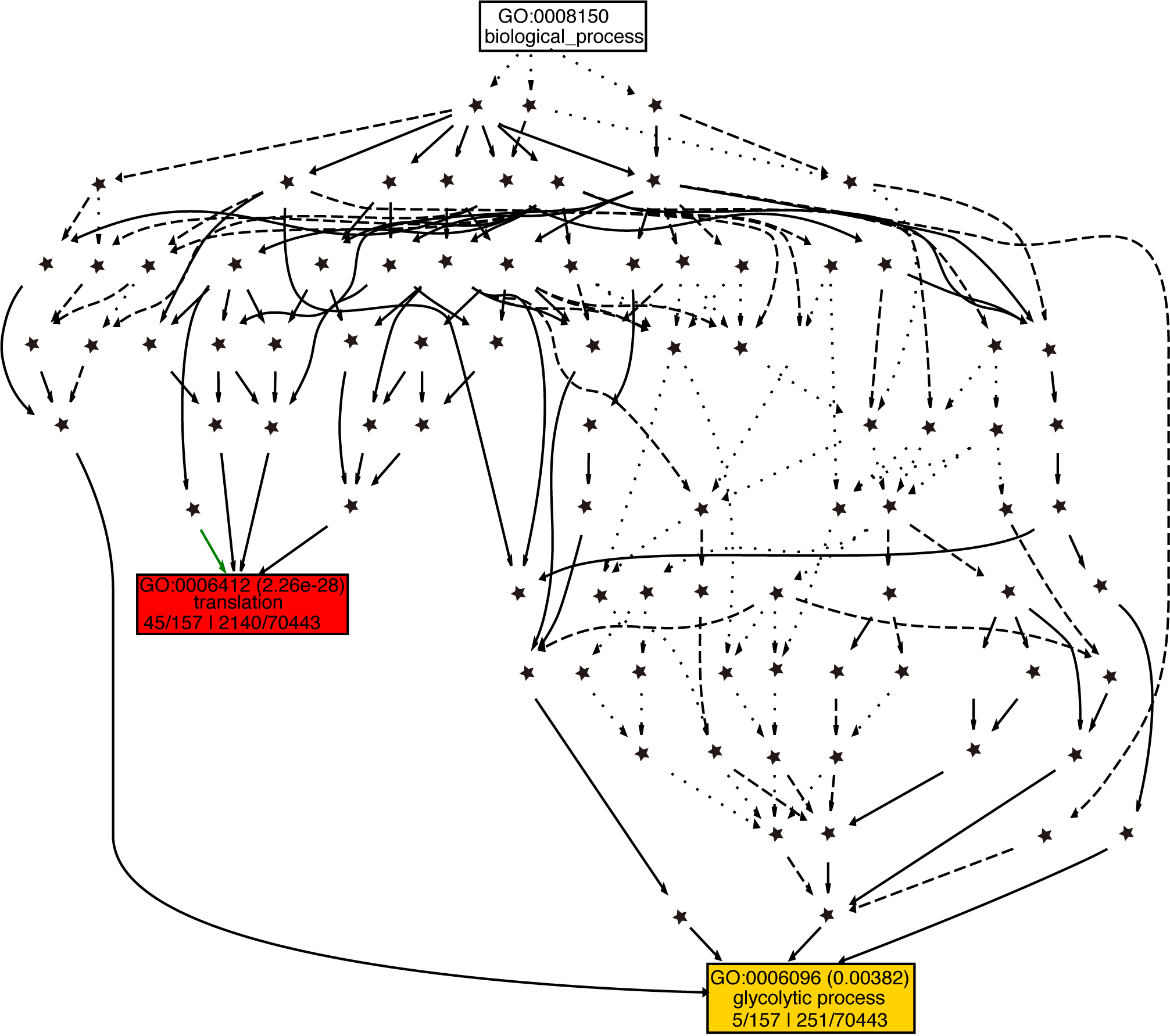

Supplement: Supplementary Figure 1 — Disease index of XH7 and XH21 after V. dahliae incubation. The number of four represented the highest disease index when the whole plant died, and the number of zero indicated the lowest disease index with no visible wilting. The numbers zero to four are also presented by different colors for visualization. [file Data_Sheet_1.zip › sFigures&Tables/Figure S7.tif]

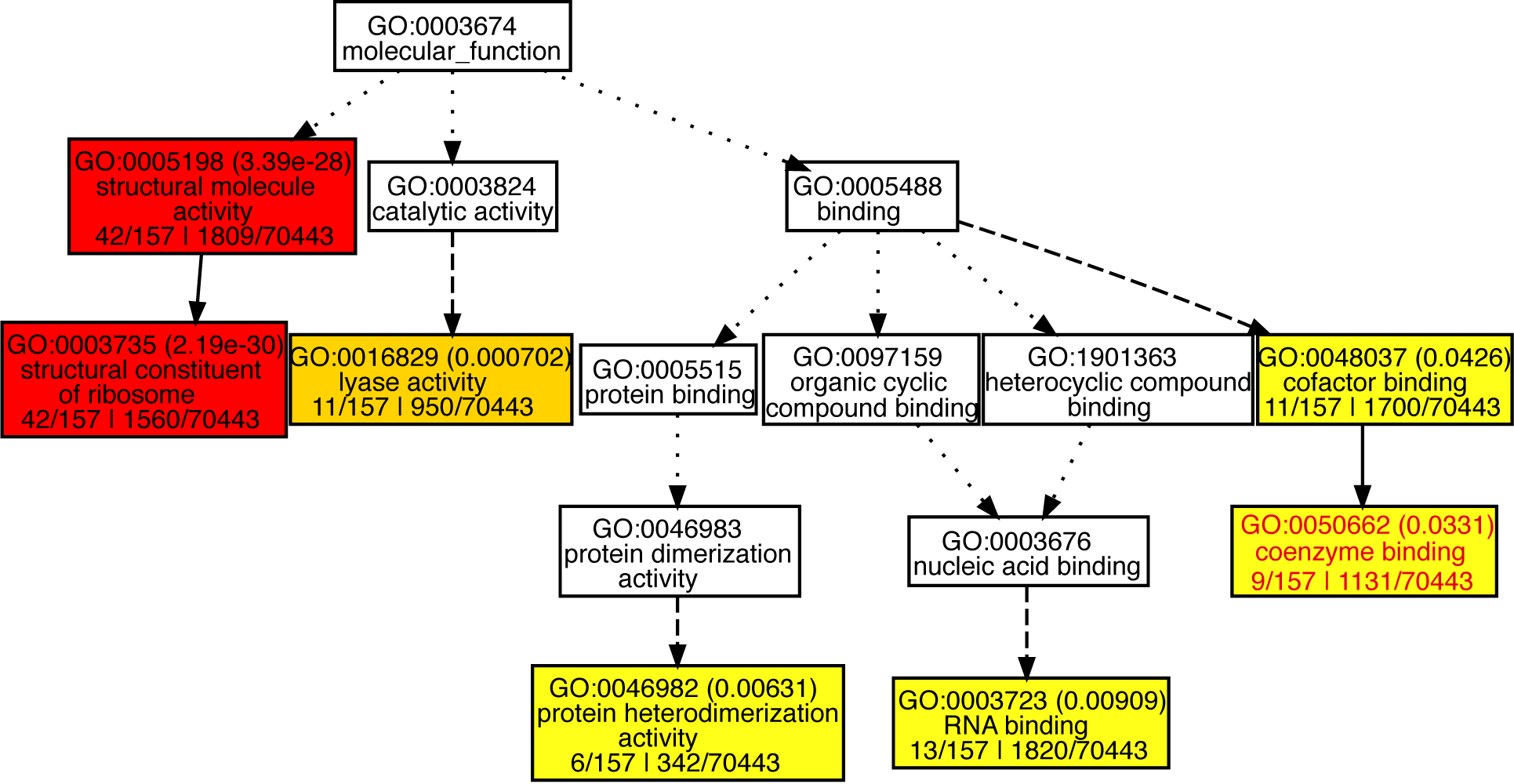

Supplement: Supplementary Figure 1 — Disease index of XH7 and XH21 after V. dahliae incubation. The number of four represented the highest disease index when the whole plant died, and the number of zero indicated the lowest disease index with no visible wilting. The numbers zero to four are also presented by different colors for visualization. [file Data_Sheet_1.zip › sFigures&Tables/Figure S6.tif]

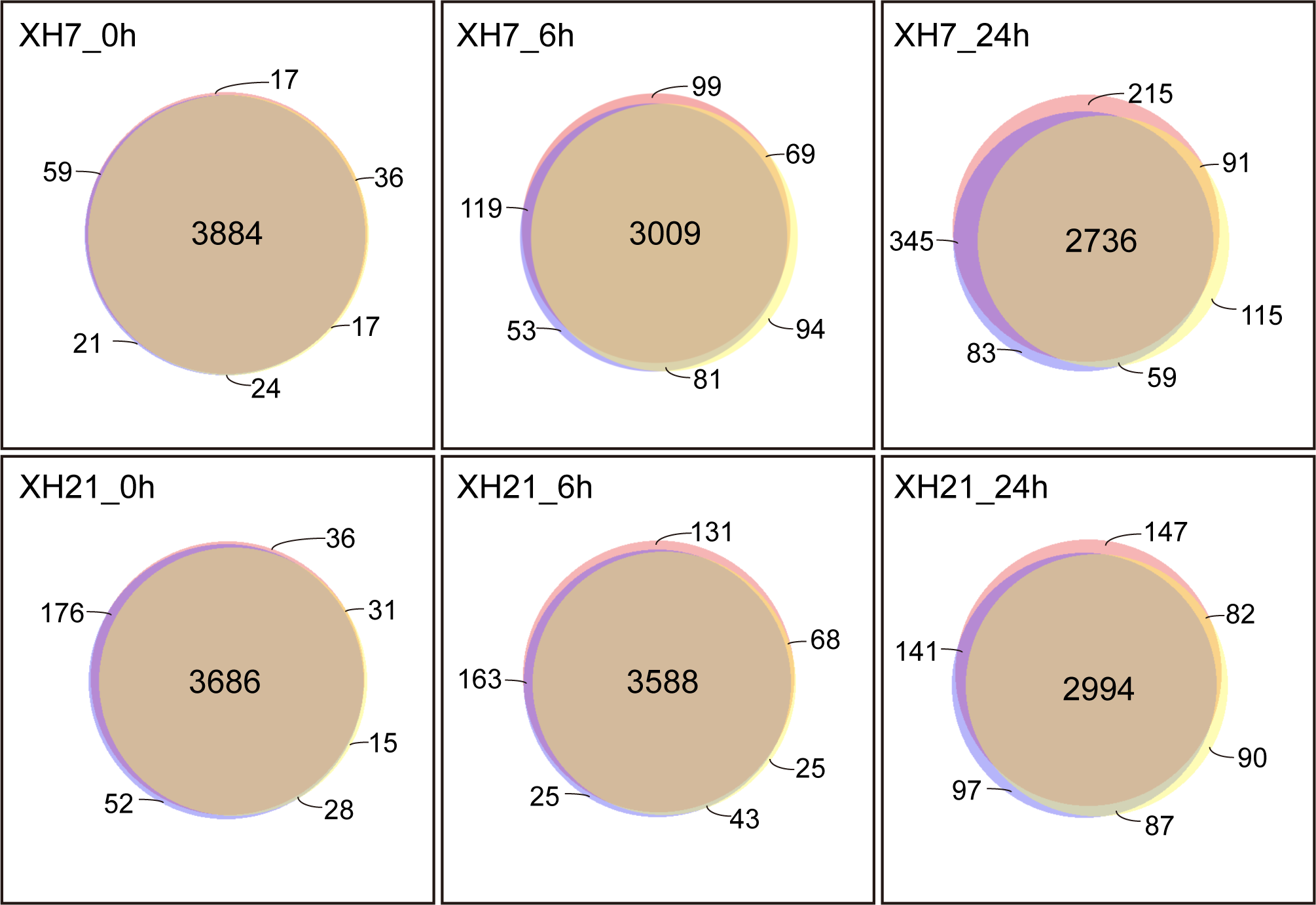

Supplement: Supplementary Figure 1 — Disease index of XH7 and XH21 after V. dahliae incubation. The number of four represented the highest disease index when the whole plant died, and the number of zero indicated the lowest disease index with no visible wilting. The numbers zero to four are also presented by different colors for visualization. [file Data_Sheet_1.zip › sFigures&Tables/Figure S2.tif]

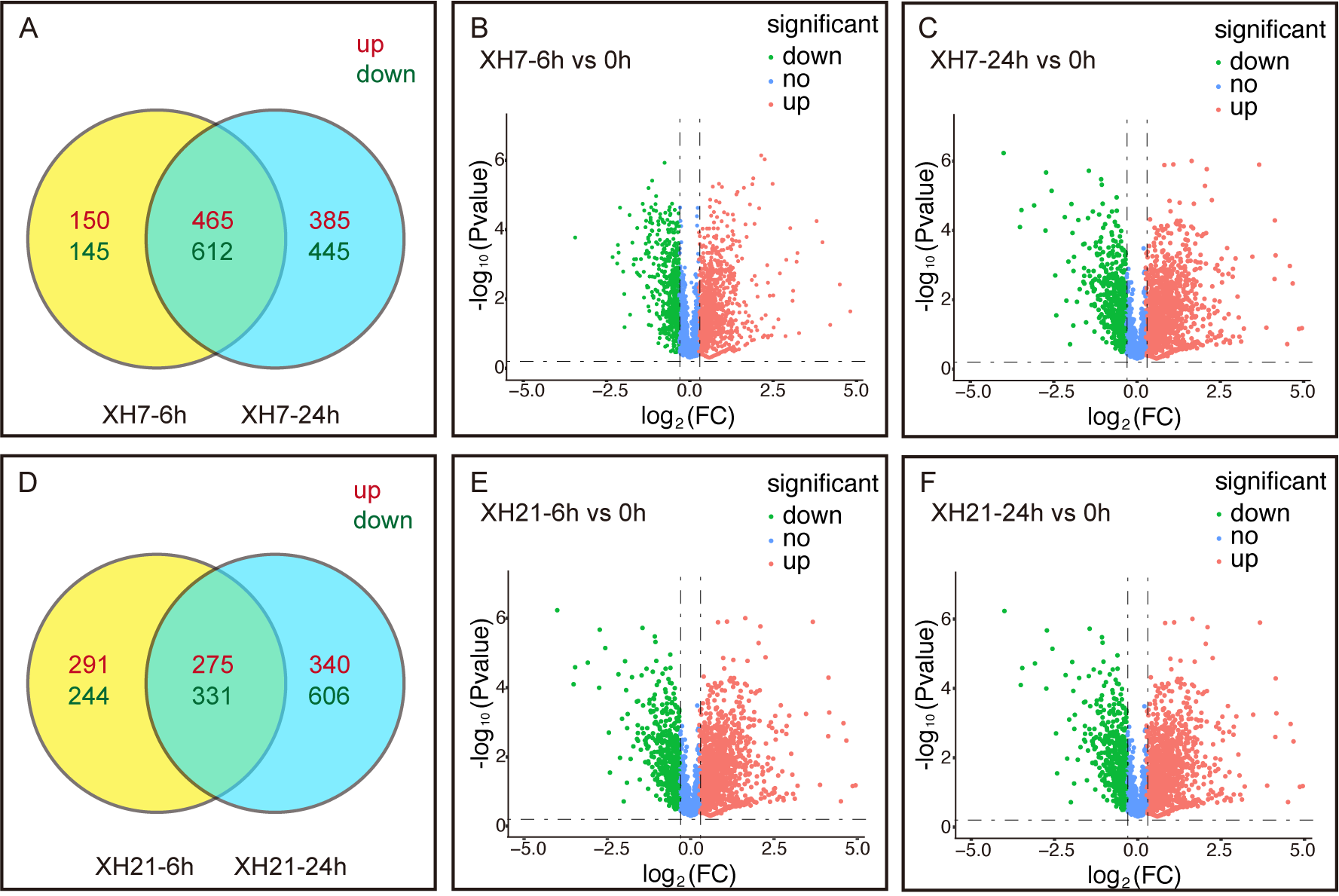

Supplement: Supplementary Figure 1 — Disease index of XH7 and XH21 after V. dahliae incubation. The number of four represented the highest disease index when the whole plant died, and the number of zero indicated the lowest disease index with no visible wilting. The numbers zero to four are also presented by different colors for visualization. [file Data_Sheet_1.zip › sFigures&Tables/Figure S3.tif]

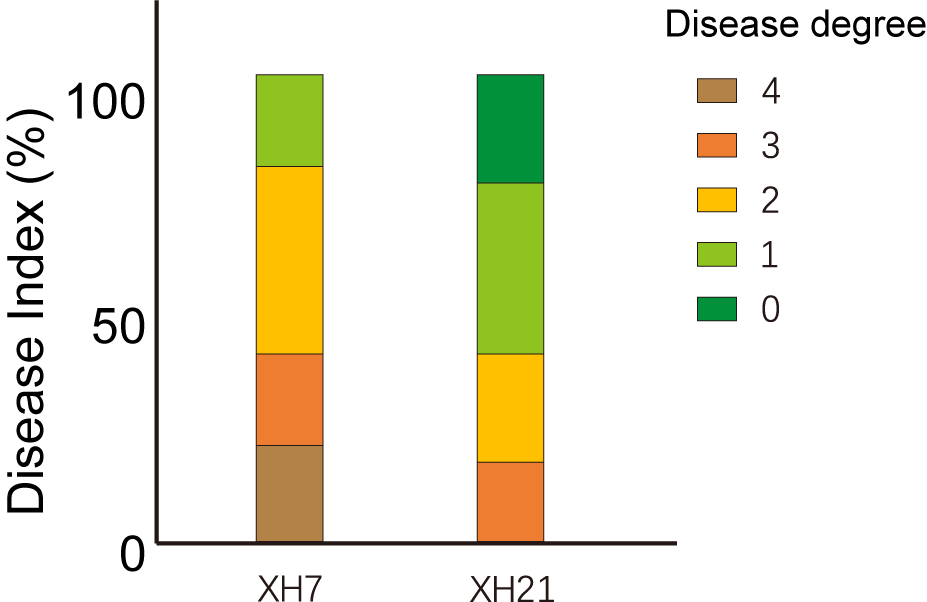

Supplement: Supplementary Figure 1 — Disease index of XH7 and XH21 after V. dahliae incubation. The number of four represented the highest disease index when the whole plant died, and the number of zero indicated the lowest disease index with no visible wilting. The numbers zero to four are also presented by different colors for visualization. [file Data_Sheet_1.zip › sFigures&Tables/Figure S1.tif]

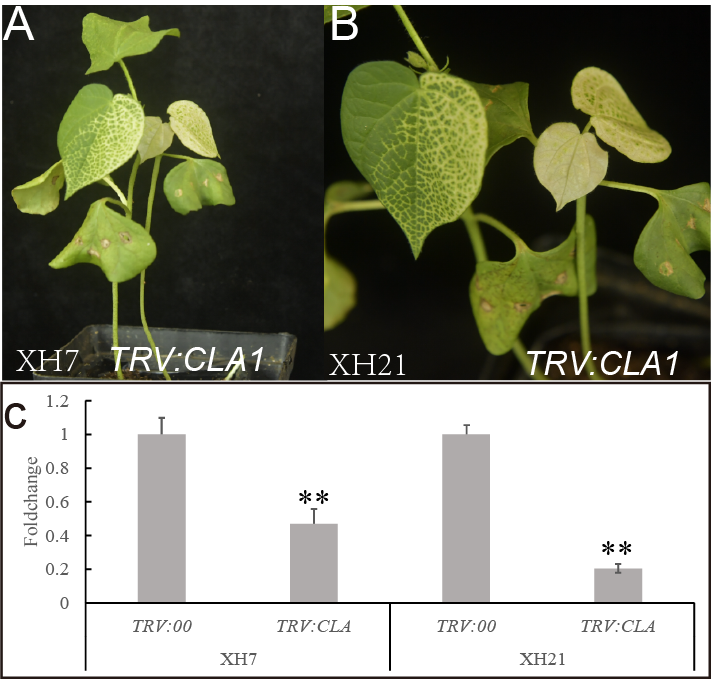

Supplement: Supplementary Figure 1 — Disease index of XH7 and XH21 after V. dahliae incubation. The number of four represented the highest disease index when the whole plant died, and the number of zero indicated the lowest disease index with no visible wilting. The numbers zero to four are also presented by different colors for visualization. [file Data_Sheet_1.zip › sFigures&Tables/Figure S12.tif]

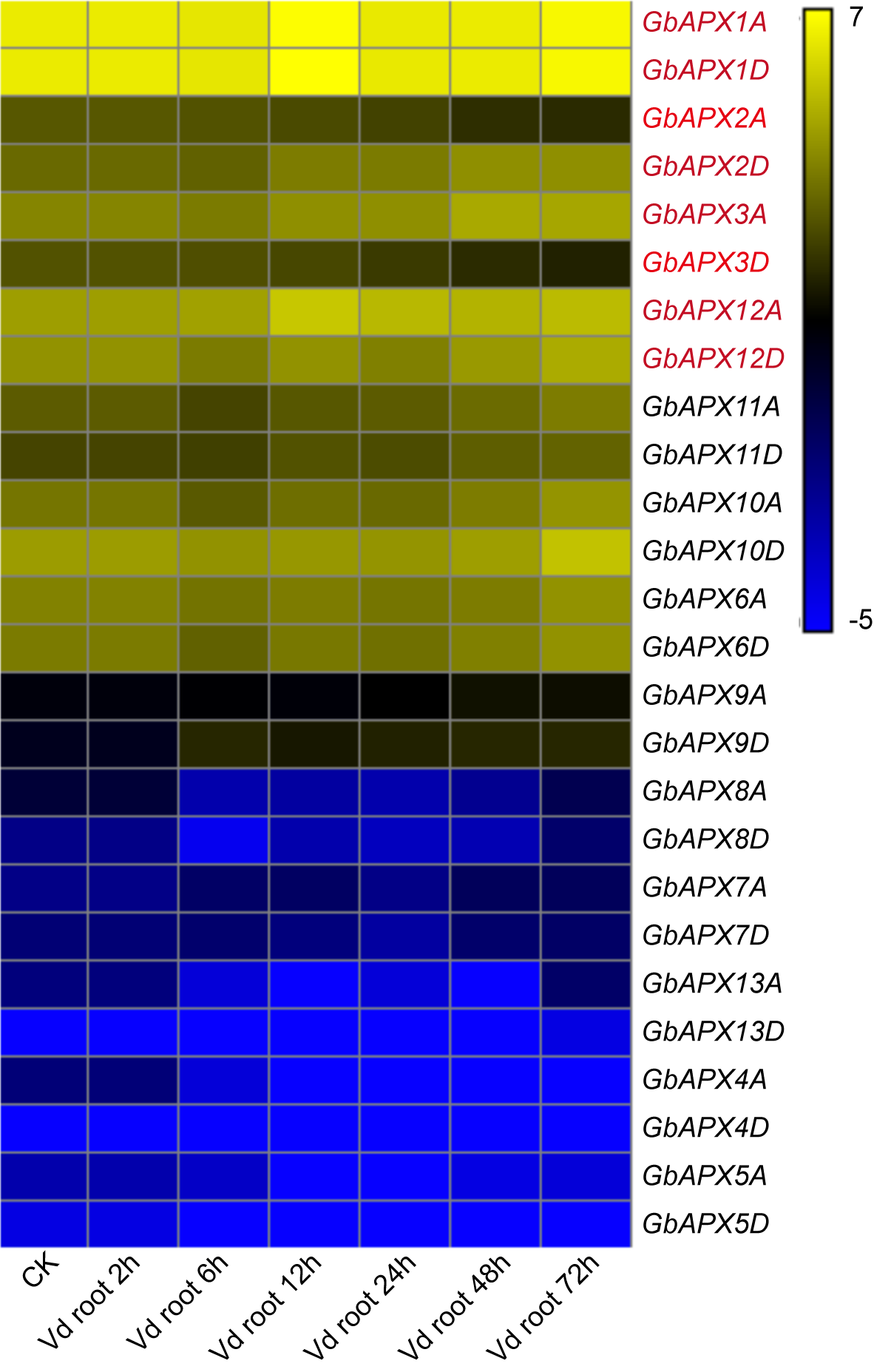

Supplement: Supplementary Figure 1 — Disease index of XH7 and XH21 after V. dahliae incubation. The number of four represented the highest disease index when the whole plant died, and the number of zero indicated the lowest disease index with no visible wilting. The numbers zero to four are also presented by different colors for visualization. [file Data_Sheet_1.zip › sFigures&Tables/Figure S11.tif]

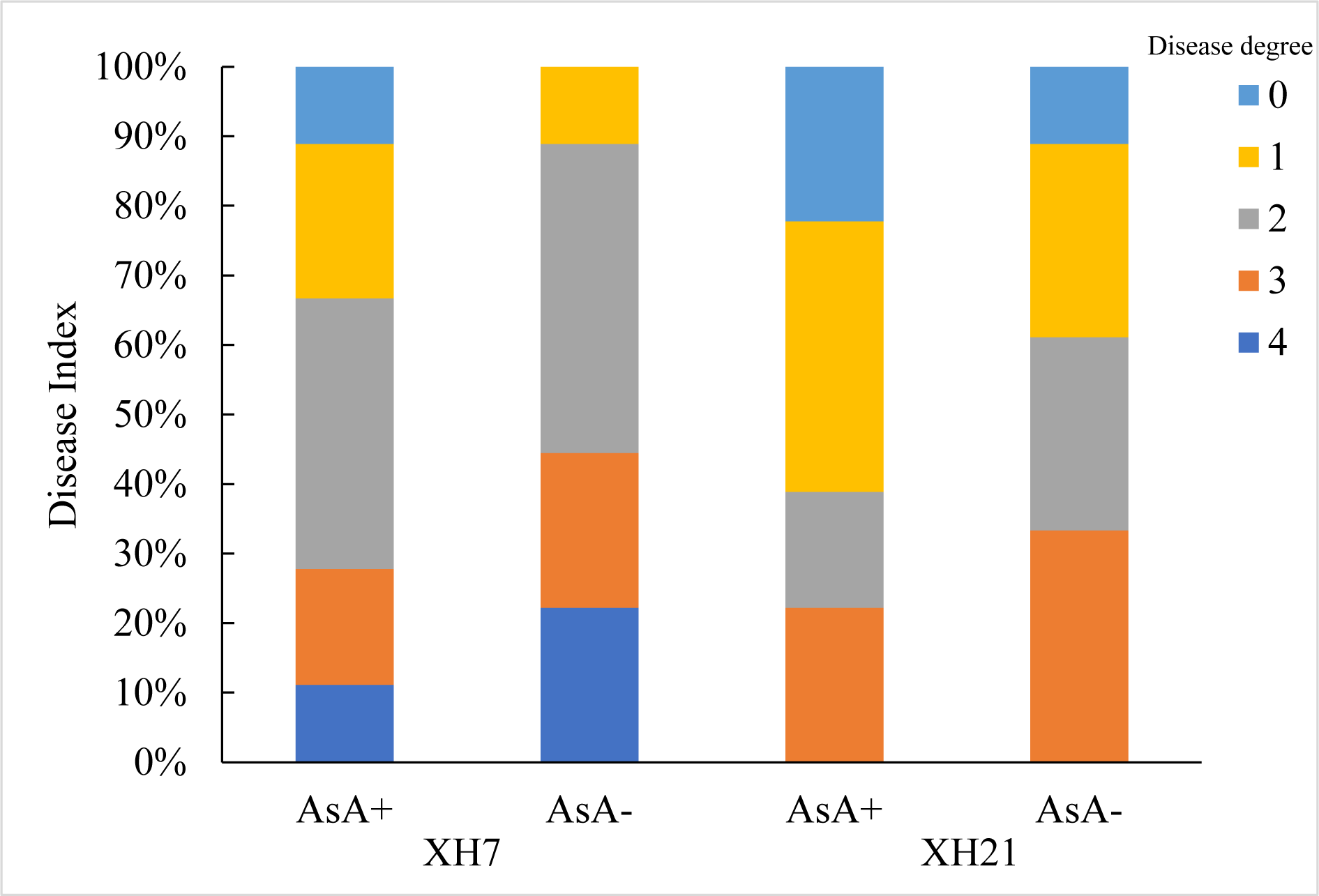

Supplement: Supplementary Figure 1 — Disease index of XH7 and XH21 after V. dahliae incubation. The number of four represented the highest disease index when the whole plant died, and the number of zero indicated the lowest disease index with no visible wilting. The numbers zero to four are also presented by different colors for visualization. [file Data_Sheet_1.zip › sFigures&Tables/Figure S10.tif]
